# Initial Post-Commercialization Experience Using a Thoracic Branch Endoprosthesis: Broad Application to Real-World Patients

**DOI:** 10.1093/ejcts/ezaf452

**Published:** 2025-12-17

**Authors:** Xiaoying Lou, Patrick R Vargo, Francis Caputo, Sean Lyden, Benjamin Kramer, Marijan Koprivanac, Vidyasagar Kalahasti, Milind Desai, Eric E Roselli, Aorta Center Investigators, Patrick Collier, Margaret Fuchs, Courtney Hanak, Nicholas Hoell, Ali Khalifeh, Levester Kirksey, Venu Menon, Jonathon Quatromoni, Bo Xu, John Mansour, Christine Jellis, Serge Harb, Leslie Cho, Jeanna Sigmund

**Affiliations:** Aorta Center, Heart, Vascular, and Thoracic Institute, Cleveland Clinic, Cleveland, OH 44195, United States; Department of Thoracic and Cardiovascular Surgery, Heart, Vascular, and Thoracic Institute, Cleveland Clinic, Cleveland, OH 44195, United States; Aorta Center, Heart, Vascular, and Thoracic Institute, Cleveland Clinic, Cleveland, OH 44195, United States; Department of Thoracic and Cardiovascular Surgery, Heart, Vascular, and Thoracic Institute, Cleveland Clinic, Cleveland, OH 44195, United States; Aorta Center, Heart, Vascular, and Thoracic Institute, Cleveland Clinic, Cleveland, OH 44195, United States; Department of Vascular Surgery, Heart, Vascular, and Thoracic Institute, Cleveland Clinic, Cleveland, OH 44195, United States; Aorta Center, Heart, Vascular, and Thoracic Institute, Cleveland Clinic, Cleveland, OH 44195, United States; Department of Vascular Surgery, Heart, Vascular, and Thoracic Institute, Cleveland Clinic, Cleveland, OH 44195, United States; Aorta Center, Heart, Vascular, and Thoracic Institute, Cleveland Clinic, Cleveland, OH 44195, United States; Department of Thoracic and Cardiovascular Surgery, Heart, Vascular, and Thoracic Institute, Cleveland Clinic, Cleveland, OH 44195, United States; Aorta Center, Heart, Vascular, and Thoracic Institute, Cleveland Clinic, Cleveland, OH 44195, United States; Department of Thoracic and Cardiovascular Surgery, Heart, Vascular, and Thoracic Institute, Cleveland Clinic, Cleveland, OH 44195, United States; Aorta Center, Heart, Vascular, and Thoracic Institute, Cleveland Clinic, Cleveland, OH 44195, United States; Department of Cardiology, Heart, Vascular, and Thoracic Institute, Cleveland Clinic, Cleveland, OH 44195, United States; Aorta Center, Heart, Vascular, and Thoracic Institute, Cleveland Clinic, Cleveland, OH 44195, United States; Department of Cardiology, Heart, Vascular, and Thoracic Institute, Cleveland Clinic, Cleveland, OH 44195, United States; Aorta Center, Heart, Vascular, and Thoracic Institute, Cleveland Clinic, Cleveland, OH 44195, United States; Department of Thoracic and Cardiovascular Surgery, Heart, Vascular, and Thoracic Institute, Cleveland Clinic, Cleveland, OH 44195, United States; Department of Thoracic and Cardiovascular Surgery, Heart, Vascular, and Thoracic Institute, Cleveland Clinic, Cleveland, OH 44195, United States; Department of Vascular Surgery, Heart, Vascular, and Thoracic Institute, Cleveland Clinic, Cleveland, OH 44195, United States; Department of Cardiology, Heart, Vascular, and Thoracic Institute, Cleveland Clinic, Cleveland, OH 44195, United States

**Keywords:** thoracic endovascular aortic repair (TEVAR), branched endoprosthesis, aortic arch aneurysm, aortic arch dissection

## Abstract

**Objectives:**

The first thoracic branched endoprosthesis (TBE) device for arch and proximal descending aortic thoracic endovascular aortic repair (TEVAR) became commercially available in the United States in 2022 (WL Gore, Flagstaff, AZ, United States). The pivotal approval study was limited to zone 2 deployment in cases of aortic aneurysm, but its use in aortic dissections and deployment in zones 0 and 1 remain investigational. We describe early results of Gore TBE device deployment in real-world patients and in a variety of clinical settings including off-label indications.

**Methods:**

Patient characteristics, procedural details, and postoperative outcomes were assessed in this retrospective single-centre cohort study. Twenty-one patients (38.2%) were not open surgical candidates due to comorbidities. Follow-up evaluations included computed tomography angiography imaging of the device and its branches in addition to out

**Patient:**

evaluation at discharge, 3 and 12 months, and then annually thereafter. The rate of endoleaks and re-interventions were assessed over the course of follow-up.

**Results:**

Between September 2022 and September 2024, 55 consecutive patients underwent aortic repair using the Gore TBE endoprosthesis at our institution. Ten (18.2%) cases were performed in an urgent/emergency setting. Indication for TBE repair was arch, descending and/or thoraco-abdominal aortic aneurysm (*N* = 26 [47.3%]), dissection (*N* = 27 [49.1%], 6 acute/subacute), intramural haematoma (*N* = 1 [1.8%]), or aortic coarctation (*N* = 1 [1.8%)]. Device deployment was performed in aortic zone 0 (*N* = 16 [29.1%]), zone 1 (*N* = 5 [9.1%]), or zone 2 (*N* = 34 [61.8%]). Twenty-four (43.6%) underwent an aortic arch debranching procedure prior to TEVAR, and 23 (41.8%) had preoperative spinal drain placement. Technical success was 100.0% following the index operation. Operative mortality was 3.6% (*N* = 2). There were no cases of stroke or paralysis and one case of renal failure requiring dialysis. One patient experienced paraparesis treated with spinal drain placement with immediate resolution. Median follow-up was 366 (interquartile range [IQR]: 189, 444) days. The rate of endoleak over the follow-up period was 16.4% (*N *= 9). Three cases were treated medically with ongoing surveillance, and the remaining 6 underwent intervention: 5 endovascular, 1 open. The linearized all-cause mortality rate was 11.0% per patient-year.

**Conclusions:**

Real-world use of the Gore TBE device demonstrates its safety and versatility in addressing various aortic pathologies and deployment zones. Further follow-up is necessary to assess the durability of repair, and novel investigational devices may improve outcomes.

## Graphical abstract

**Figure:**
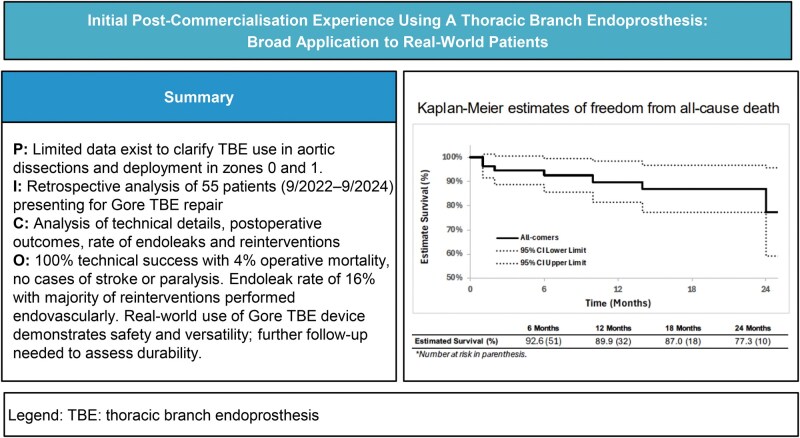

## Introduction

Since its commercial approval for zone 2 disease in May 2022, the GORE TAG thoracic branch endoprosthesis (TBE: W.L. Gore, Flagstaff, AZ) has expanded endovascular treatment options for arch and proximal descending aortic repair by maintaining side branch (SB) patency.^1^ The modular device consists of a main body thoracic aorta stent graft with a single SB portal and a mating SB component composed of spiral nitinol stents covered with expanded polytetrafluoroethylene fabric.^1^

The recent pivotal study was limited in its use to cases of aortic aneurysms meeting strict anatomic criteria for zone 2 deployment.^2–4^ Zone 2 deployment in the setting of blunt thoracic aortic injury^5^ and acute aortic syndrome^6^^,^^7^ has also been described. However, its use for aortic dissections and proximal lesions deployed in zones 0 and 1 was investigational with limited data until very recently.^8–10^ The primary objective was to assess the use of the GORE TAG TBE for various thoracic aortic pathologies in real-world patients since initial commercial approval and provide early and mid-term outcomes. We describe patient characteristics and procedural details and demonstrate its utility, flexibility, and application in a variety of clinical settings.

## Methods

This was a retrospective single institution experience with use of the GORE TBE device to treat consecutive patients presenting with thoracic aortic disease (aneurysm or dissection) between September 2022 and September 2024 who were determined eligible for treatment. Deployment zones in the present analysis are based on the most proximal extent of graft deployment as referenced by Ishimaru aortic zones. This study adheres to the STROBE guidelines.

### Study design and population

All patients were evaluated by the aortic team at the Cleveland Clinic comprised of cardiologists, cardiac imaging specialists, cardiac surgeons, and vascular surgeons to assess surgical risk and evaluate anatomical inclusion for device consideration. Comorbidities considered high risk for open heart surgery and favouring endovascular intervention included age ≥80 years old, prior median sternotomy, severe chronic obstructive pulmonary disease, chronic kidney disease ≥3 b, and poor baseline functional status. Primary end-points included technical success and absence of serious adverse events: operative mortality (ie, in-hospital mortality and mortality ≤30 days of the index operation), aortic rupture, disabling stroke as characterized by changes in the modified Rankin score, permanent paraplegia or paraparesis, and new-onset renal failure requiring dialysis. Device technical success was defined as successful access and delivery of device components to intended sites, retrieval of delivery systems, patency of grafts, and the absence of unanticipated additional procedures related to the device system.^2^

### Device and procedure

A description of the TBE device and its deployment in zone 2 has been published previously.^1^ The 2 primary system components consist of the aortic component (AC) and SB component which is deployed within a distally oriented portal within the AC (**Supplementary Figure**). The AC is tracked over a stiff wire (Lunderquist; Cook Medical Inc, Bloomington, IN, United States), and the SB is delivered over a through-and-through access wire (Metro; Cook Medical Inc, Bloomington, IN, United States) introduced via the right or left brachial artery. Following SB deployment, a large compliant balloon (Coda; Cook Medical Inc, Bloomington, IN, United States) is used to expand the SB within the AC portal to optimize full expansion and seal. Further ballooning of the AC is undertaken as needed.

Additional aortic stent grafts may be deployed proximally to improve AC seal. In residual Debakey I or Debakey III dissection cases, we typically extend covered stent grafts (CTAG; WL Gore, Flagstaff, AZ) to the level of the coeliac axis and in those with thoraco-abdominal involvement of the dissection and without aneurysmal changes limited to the thoracic aorta, additional bare metal stenting (Zenith Dissection Stent, Cook Medical Inc, Bloomington, IN) is extended to the level of the aortic bifurcation.^11^ In cases of chronic dissection, balloon fracture fenestration is also performed through the covered portion of repair to further expand the true lumen and promote favourable aortic remodelling.^12^

In cases of zone 1 or 0 deployment, surgical revascularization of the left subclavian artery (LSA, zone 1) or both the LSA and left common carotid artery (LCCA, zone 0) via extra-anatomic bypass or native vessel transposition is first performed as a staged operation. For debranching to facilitate zone 0 deployment, we routinely utilize cerebral oximetry and maintain mean arterial pressures of 80-100 mmHg. If cerebral oximetry shows a fall in saturations of >20.0% from baseline, we employ a shunt to support cerebral blood flow intraoperatively. The second staged TBE deployment is typically performed at least 1 day after and within a week of the first stage debranching. Vessel ligation (during first stage) or endovascular occlusion (during second stage) of the excluded aortic arch branch vessels is also performed as part of the repair to prevent type II endoleaks.

All cases in this series were performed jointly by an aortic-trained cardiac and vascular surgeon. Preoperative workup included computed tomography angiography (CTA) imaging with three-dimensional multi-planar reconstruction (TeraRecon Inc, Durham, NC, United States) to assist with operative planning and device sizing. Preoperative lumbar drain was utilized for those requiring extensive full thoracic aortic coverage and considered high risk for spinal cord ischaemia (eg, prior abdominal repair, significant peripheral arterial disease). All patients underwent general endotracheal anaesthesia. Heparin was administered at a dose of 100 units/kg for an ACT goal >250 seconds throughout the duration of the case. All patients were extubated in the operative room with full neurovascular assessment prior to transfer to the intensive care unit.

### Follow-up

Follow-up evaluations included multi-phase CTA imaging of the device and its branches in addition to outpatient evaluation at discharge, 3 months, and 12 months, and then annually thereafter. The rate of endoleaks and re-interventions was assessed over the course of follow-up.

### Statistical methods

Standard statistical methods were used for data analysis and presentation including mean (SD [standard deviation]) for normally distributed continuous variables, median (interquartile range [IQR]) for non-normal data, and *N* (%) for binary or categorical data. Time to event data were analysed and represented using the Kaplan-Meier reverse method to account for censoring and all reported estimates had standard errors <0.1. The follow-up interval for each patient was defined from the date of the index operation to time of death or the end of the study period, whichever occurred first.

The Cleveland Clinic Institutional Review Board (IRB# 24-048) waived the need for individual patient consent. This study conforms to all ethical principles as specified by the Declaration of Helsinki.

## Results

### Baseline characteristics

Patient demographics and clinical characteristics at baseline are provided in **Table 1**. Of the 55 patients included in the analysis, a majority were men (*N* = 37, 67.3.), and the mean age was 68 (SD = 15) years. A significant proportion of patients presented with a history of stroke (*N* = 14, 25.5%) and chronic obstructive pulmonary disease (*N* = 15, 27.3%). Seven patients (12.7%) had a diagnosis of heritable thoracic aortic disease at presentation: 6 Marfan syndrome, 1 with extensive family history but genetic mutations of unknown significance. Most patients (*N* = 31, 56.4%) had a history of prior open aortic surgery. By consensus of the aortic team, 38.2% were referred for TBE deployment because they were deemed prohibitive risk for open surgical repair.

**Table 1.** Patient and Aortic Pathology Details

*Patient characteristics*	
Age, years	68 ± 15
Male gender	37 (67.3%)
BMI	28 ± 6
Comorbidities	
Hypertension	49 (89.1%)
Diabetes	6 (10.9%)
Hypercholesterolaemia	35 (63.6%)
Coronary artery disease	8 (14.5%)
Peripheral artery disease	2 (3.6%)
Previous stroke	14 (25.5%)
Chronic obstructive pulmonary disease	15 (27.3%)
Hereditary thoracic aortic disease	7 (12.7%)
History of open aortic surgery	31 (56.4%)
Not an open surgical candidate	21 (38.2%)
*Aortic pathology characteristics*	
Aneurysm	26 (47.3%)
Location	
Ascending aorta	8
Arch	9
Descending aorta	9
Length of lesion (mm)	38 (5, 140)
Maximum diameter (mm)	56 (36, 81)
Dissection	27 (49.1%)
Chronicity	
Acute/subacute	6
Chronic	21
Classification	
Debakey I (residual)	14
Debakey III	13
Intramural haematoma	1 (1.8%)
Aortic coarctation	1 (1.8%)

Values are mean ± standard deviation, counts (%), or median (range lowest, highest).

Abbreviation: BMI: body mass index.

### Aortic pathology/indications

Indications to proceed with TBE deployment were evenly split for degenerative aortic aneurysm and dissection aetiologies (**Table 1**). For those presenting with degenerative aneurysms (*N* = 26, 47.3%), the median maximum aortic diameter was 56 (IQR: 36, 81) mm, and the length of the lesion was 38 (5140) mm. Among cases of aortic dissection (*N* = 27, 49.1%), most patients (*N* = 21) presented in the chronic phase, and there was a similar distribution of residual Debakey I versus Debakey III patients.

### Procedural details

Most cases were elective (*N* = 45, 81.8%), but 10 patients (18.2%) underwent thoracic endovascular aortic repair (TEVAR) at index hospitalization for urgent concerns of impending rupture or emergency malperfusion (**Table 2**). Device deployment was performed in aortic zone 0 (*N* = 16, 29.1%), zone 1 (*N* = 5, 9.1%) or zone 2 (*N* = 34, 61.8%) (**Table 2**). Preoperative lumbar drain insertion was utilized in 23 patients (41.8%). All patients underwent percutaneous brachial artery access, and a majority (72.7%) were performed with percutaneous femoral access.

**Table 2.** Operative Details

	All-comers (*n* = 55)
Status	
Elective	45 (81.8%)
Urgent/emergency	10 (18.2%)
Arch debranching utilized	24 (43.6%)
Zone 0 Deployment	16 (29.1%)
Zone 1 Deployment	5 (9.1%)
Zone 2 Deployment	34 (61.8%)
Spinal drain utilized	23 (41.8%)
Percutaneous access	40 (72.7%)
Fluoroscopy duration (min)	33 ± 22
Contrast volume (mL)	95 ± 63
Median additional stent grafts deployed distal to AC	1 (0, 4)
Dissection stent deployed distal to AC	18 (32.7%)
AE cuff needed	5 (9.1%)

Values are mean ± standard deviation, counts (%), or median (range lowest, highest).

Abbreviation: AC: aortic component; AE: aortic extender.

Aortic debranching was utilized in 24 patients (43.6%). Three zone 2 patients underwent LSA debranching due to high risk of LCCA compromise. Two patients had a history of residual dissection with aneurysmal degeneration and underwent LCCA-LSA bypass to facilitate a sufficient proximal landing zone. The third presented with distal arch aneurysm and underwent LCCA-LSA transposition with ligation of an anomalous left vertebral artery.

All patients undergoing zone 0 (*N* = 16) or zone 1 (*N* = 5) deployment required more extensive debranching as follows. Of zone 0 patients, 3 debranching strategies were utilized. Two patients had a prior sternotomy with arch vessel debranching off their innominate artery, 4 underwent cervical debranching with posterior bypasses, and 10 underwent manubriotomy with anterior aortic arch debranching. The SB component was deployed into the innominate artery for all but one patient. This patient had a complex aortic arch pseudoaneurysm with residual dissection flaps in the innominate and LSA arteries. She was not a candidate for open repair. This was treated with LCCA-RSA bypass and RCCA transposition onto the bypass followed by zone 0 TBE with SB deployment into the LCCA as well as false lumen embolization of the innominate artery and LSA.

Of zone 1 patients, 2 underwent cervical debranching with LCCA-LSA bypass or transposition with SB deployment into the LCCA and coverage of the LSA ostium. The other 3 patients underwent debranching with LCCA ligation at its base via either a cervical approach or a manubriotomy. This technique created sufficient proximal landing zone to facilitate SB deployment into the LSA with coverage of the LCCA ostium.

The most frequent deployment configuration was one main body TBE with a single SB and concomitant distal covered stent TEVAR extension. Eighteen patients (32.7%) underwent extension of covered stenting to zone 5 and dissection stenting to zone 9, and 5 patients (9.1%) needed further proximal extension with aortic extension (AE) cuffs.

### Operative outcomes

TBE deployment was technically successful in 100.0% of patients (**Table 3**). There were no issues with device deployment or SB patency at the conclusion of the procedure or over the course of follow-up.

**Table 3.** Postoperative Outcomes

	All-comers (*n* = 55)
Technical success	55 (100.0%)
Operative mortality	2 (3.6%)
Stroke	0
Prolonged intubation >72 hours	3 (5.5%)
Renal failure requiring dialysis	1 (1.8%)
Myocardial infarction	0
Paraparesis (temporary)	1 (1.8%)
Paraplegia	0
Retrograde type A	1 (1.8%)
Aortic rupture	1 (1.8%)
Access site complication	3 (5.5%)
1 year mortality	5 (9.1%)
Overall mortality	8 (14.5%)
Reinterventions	
Second-stage (planned) open thoraco-abdominal replacement	4
Infection	1
Endoleak	6

Values are counts or counts (%).

Operative mortality was 3.6% (*N* = 2). Neither patient was a candidate for open aortic surgery. The first had a history of multiple prior aortic operations and severe COPD who underwent zone 0 TBE deployment for aortic arch pseudoaneurysm. While the intraoperative course was uneventful, he developed acute-onset chest pain and abdominal distension on postoperative day 1 with haemodynamic deterioration. Imaging demonstrated a new re-entry tear and active contrast extravasation originating from the descending aorta. He was taken for emergency TEVAR extension with exploratory laparotomy but developed multi-organ failure. His family elected to withdraw care. The other patient was an octogenarian who underwent zone 2 TBE deployment for a saccular distal aortic arch aneurysm. Intraoperative course was uncomplicated, and completion angiogram did not reveal any concerns. Routine postoperative CT scan demonstrated a retrograde type A aortic dissection. She was readmitted to the intensive care unit for impulse control and taken to the operating room, where trans-oesophageal echo demonstrated a stable dissection with thrombosed proximal false lumen. Given her frail and deconditioned state, this was managed medically. She ultimately died in the hospital of respiratory failure from pneumonia 31 days postoperatively.

There was one case of transient neurological deficit with a negative CT of the brain and complete resolution of neurological deficits by time of hospital discharge. There were no cases of stroke or paraplegia. One patient experienced paraparesis that resolved by time of hospital discharge after lumbar drain insertion and permissive hypertension.

**Figure 1.**
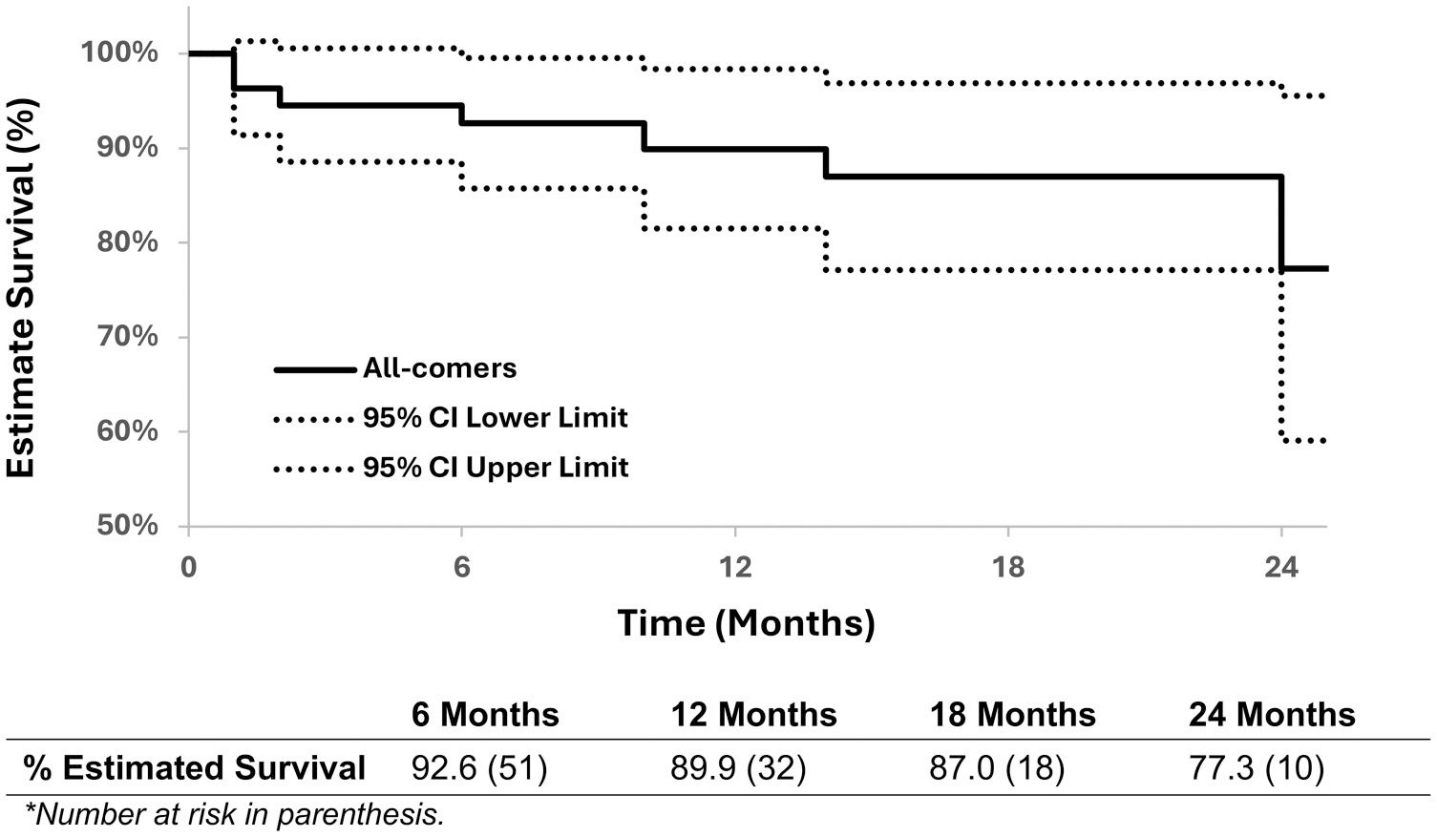
Kaplan-Meier Estimates of Freedom From All-Cause Death.

All-cause mortality over our follow-up period was 14.5% (*N* = 8) with estimated 89.9% (95% CI: 81.5%, 98.3%) survival at 1 year (**Figure 1**). The linearized mortality rate was 11.0% per patient-year. Exact cause of death was not ascertainable in our analysis, but available documentation for mortalities occurring after hospital discharge were not aortic-related.

### Endoleaks and reinterventions

Eleven patients underwent reintervention over the course of follow-up with timing and outcomes as listed in **Table 4**. Four underwent planned second-stage open thoraco-abdominal aortic replacements with no postoperative mortalities. One patient re-presented with a newly diagnosed ascending aortic pseudoaneurysm almost 2 years after initial TBE deployment. The patient had a history of multiple prior open aortic surgeries and was not deemed to be an open surgical candidate. He underwent successful coil embolization with resolution of the pseudoaneurysm.

**Table 4.** Endoleaks and Negative Remodelling

Patient	Endoleak type	Location of leak	Reintervention	Treatment	Timing of reintervention (days postoperative)	Outcome
1—Aneurysm	II	Zone 3	No	Surveillance	N/A	Resolution of endoleak
2—Aneurysm	II	Zone 3	No	Surveillance	N/A	Ongoing follow-up with stability at 5 months
3—Aneurysm	Ia	Zone 0-1	No	Surveillance	N/A	Ongoing follow-up with stability at 2 months^a^
4—Dissection	RFLP	Zone 4-5	Yes, endovascular	Coil embolization of intercostals and FL	4	Progressive aneurysmal degeneration of thoraco-abdominal aortic aneurysm^a^
5—Dissection	RFLP	Zone 2-3	Yes, endovascular	Left subclavian artery FL embolization	18	Resolution of endoleak
6—Dissection	RFLP and III	Zone 2-5	Yes, endovascular	TEVAR re-lining and left subclavian FL embolization	42	Resolution of endoleak from left subclavian; Persistent type II leak and aneurysmal degeneration in zones 5-8 and may need open repair
7—Dissection	1a	Zone 0-1	Yes, open	Open arch replacement using reverse FET technique	43	Resolution of endoleak
8—Dissection	1a and RFLP	Zone 2-5	Yes, endovascular	TEVAR proximal extension and left subclavian FL embolization	46	Resolution of endoleak
9—Dissection	RFLP	Zone 4-5	Yes, endovascular	Coil embolization of intercostals and FL	121	Resolution of endoleak

a N/A - Not a candidate for any further open or endovascular therapies.

Abbreviations: FL: false lumen; FET: frozen elephant trunk; RFLP: retrograde false lumen perfusion.

**Figure 2.**
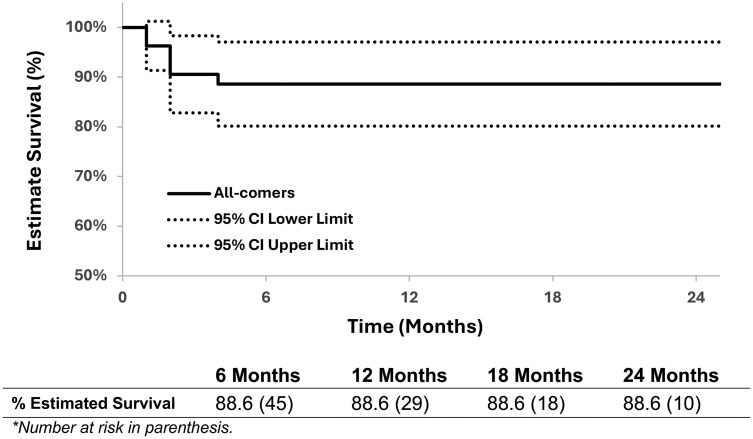
Kaplan-Meier Estimates of Freedom From Aortic Reintervention for Endoleak or Negative Remodelling.

Nine patients were diagnosed with endoleaks, most frequently within 3 months of their index operation (**Table 4**). Three were surveilled with resolution or stability. Of the 6 patients who underwent reintervention for endoleak over the follow-up period, 5 were treated endovascularly and 1 with open arch replacement. The majority of endoleaks resolved following reintervention. Freedom from reintervention for endoleak or negative aortic remodelling was 88.6% (95% CI: 80.2%, 97.1%) at 1 year (**Figure 2**). The linearized reintervention rate was 10.3% per patient-year.

### Follow-up

Median follow-up for our study cohort was 366 (IQR: 189, 444) days. Twenty-nine (52.7%) patients had follow-up imaging of at least 1 year and 44 (80.0%) at least 6 months.

## Discussion

### Principle findings

This series builds on the initial TBE pivotal study data demonstrating safety and efficacy at 12 months of follow-up in 84 patients with zone 2 aortic aneurysms.^1–3^ This real-world post-commercial experience highlights the safety and versatility of using the Gore TBE device in treating a wide range of aortic pathologies and indications. There was a roughly even split of aneurysm and dissection patients, and 18.2% were performed for urgent/emergency indications. A surprisingly large proportion of device deployments were in zone 0 and zone 1 in conjunction with aortic debranching despite our centre performing over 140 open total arch replacements annually with excellent outcomes.^13^

A variety of debranching strategies are tailored to each patient’s anatomy and aortic pathology. For zone 0 treatment, we favour an anterior arch transposition approach performed via manubriotomy. This can be challenging in the redo setting or if aneurysm is densely adherent to the posterior sternum, but it may minimize the risk of dysphagia that can complicate the cervical bypass approach. Most zone 1 patients were successfully debranched cervically with LCCA-LSA bypass or transposition. In some situations, the zone 0 or 1 arch debranching may be based off the LCCA or LSA depending on the patient’s anatomy and pathology. In a case with severe innominate artery disease, the debranching can be re-routed to the LSA allowing for the use of a commercially available 10 cm CTAG device as proximal extension. Although they are currently investigational, site specific ascending stent graft devices (ASG; WL Gore, Flagstaff, AZ) show promise to further expand options for modular treatment.^14^

Expanding TEVAR into the arch is a known risk for retrograde type A dissection and stroke.^15^^,^^16^ One case of retrograde type A dissection occurred early in the series and contributed to that patient’s death. Neurologic outcomes were excellent in this series, and the results compare favourably to literature for both elective^2^^,^^3^ and emergency cases^4^^,^^5^ of zone 2 TBE deployment and other series of arch branch devices.^17–21^ This may be attributable to the large population of chronic dissection patients and selective imaging screening to avoid some of the most severe cases of arch disease with mobile-appearing atheroma. We also use cerebral oximetry monitoring during debranching procedures and limit device manipulation in the arch.

The recently published detailed analysis of stroke events from the pivotal trial among 238 patients undergoing zone 2 TBE deployment reported stroke rate of 2.5% at 30 days and 5.9% through 12 months.^16^ In this cohort, there were no dissections of the LSA and no loss of patency of the SB component. There was an increased rate of stroke among patients with aortic aneurysm pathology. The authors theorized this may be related to more thrombus and atherosclerosis in these patients and the need for wire and catheter manipulation within the arch.^16^

There is a slight learning curve to TBE deployment. Obtaining through-and-through wire access and managing the subsequent wire wrap around the device requires are unique to this type of device.^1^^,^^17^ While advancing the AC component, tension is maintained on the through-and-through wire and rotational alignment is achieved by positioning the SB portal towards the greater curve of the aorta. If wire wrap occurs, the system is withdrawn to the descending aorta and untwisted before re-advancement. Performing these manoeuvres in the descending aorta provides some safety but the need for additional wire and device manipulation may increase the risk for embolization from the arch.

Despite an endoleak rate of 9.8%, the reintervention rate at 30 days after discharge and at mid-term follow-up remained <2.0%^3^^,^^10^ in the pivotal studies of TBE. In our real-world study with a diverse population, we observed similar trends among patients with aneurysm. Only 3 patients in this group developed an endoleak, and none have required reintervention during follow-up. There was a higher need for reintervention amongst patients with dissection pathology and endoleak or negative remodelling (*N* = 6): one underwent open surgery, and the rest were treated with an endovascular approach (**Table 4**). Of the 7 patients with known hereditary thoracic aortic disease (HTAD), 3 required further intervention after TBE for open thoraco-abdominal aortic replacement. As with any patient being treated for multi-segment aortic disease, particularly dissection cases and those with a known hereditary aortic disease, it is critical to follow all patients with vigilant surveillance imaging to assess for endoleaks, device integrity, and disease progression.

Our analysis is subject to the limitations of any retrospective single institution analysis. The sample size did not allow for comparisons between groups stratified by pathology or TBE deployment zones. Similarly, given the complex and heterogenous case presentations of this cohort, we cannot identify risk factors predictive of adverse events. While over half of all patients had follow-up over a year, ongoing surveillance will be necessary to assess the durability of our results. Further innovations in endovascular aortic repair include the development of site-specific components for the ascending aorta, and we anticipate the ability to combine these devices to treat more patients with aortic pathologies involving more proximal zones.^14^ These promising results with the TBE device continue to expand the utility of endovascular techniques for high-risk, high-complexity aortic patients.

## Conclusions

Thoracic single-branched endovascular repair is safe and versatile in addressing various aortic pathologies across each of the arch deployment zones. Although there is a need for extensive aortic debranching strategies particularly to address complex proximal aortic pathology, its versatile deployment configurations permit anatomic and disease-specific repairs. Early follow-up demonstrated a limited risk of endoleak and need for aortic reinterventions. Further follow-up is needed to assess the durability of repair.

## Supplementary Material

ezaf452_Supplementary_Data

## Data Availability

Data underlying this article will be shared on reasonable request to the corresponding author.
