# Supplementary material for: Initial Post-Commercialization Experience Using a Thoracic Branch Endoprosthesis: Broad Application to Real-World Patients
Source: Eur J Cardiothorac Surg. 2025 Dec 17;68(1):ezaf452. doi: 10.1093/ejcts/ezaf452 (PMC12798527; doi:10.1093/ejcts/ezaf452)
Supplement: ezaf452_Supplementary_Data [file ezaf452_supplementary_data.zip › Supplementary Table 1.pdf]

Supplementary Table 1. Baseline characteristics and outcomes based on aortic pathology (aneurysm vs dissection)*

	All-comers (n=55)	Aneurysm (n=26)	Dissection (n=27)
**Patient characteristics**			
Age, years	68±15	70±14	64±12
Male gender	37	15	21
BMI	28±6	27±4	29±7
Comorbidities			
Hypertension	49	23	25
Diabetes	6	5	0
Hypercholesterolemia	35	16	17
Coronary artery disease	8	3	5
Peripheral artery disease	2	0	2
Previous stroke	14	7	6
Chronic obstructive pulmonary disease	15	9	6
Hereditary thoracic aortic disease	7	1	6
History of open aortic surgery	31	13	18
**Operative outcomes**			
Technical success	55	26	27
Operative mortality	2	2	0
Stroke	0	0	0
Prolonged intubation >72 hrs	3	2	1
Renal failure requiring dialysis	1	1	0
Myocardial infarction	0	0	0
Paraparesis (temporary)	1	1	0
Paralysis	0	0	0
Retrograde type A	1	1	0
Aortic rupture	1	1	0
Access site complication	3	2	1
1 year mortality	5	3	2
Overall mortality	8	4	4

BMI. Body mass index.

Values are mean±standard deviation or counts.

*Excluding IMH (n=1) and coarctation (n=1) pathologies.
