# Supplementary material for: Initial Post-Commercialization Experience Using a Thoracic Branch Endoprosthesis: Broad Application to Real-World Patients
Source: Eur J Cardiothorac Surg. 2025 Dec 17;68(1):ezaf452. doi: 10.1093/ejcts/ezaf452 (PMC12798527; doi:10.1093/ejcts/ezaf452)
Supplement: ezaf452_Supplementary_Data [file ezaf452_supplementary_data.zip › Supplementary Table 2.pdf]

Supplementary Table 2. Baseline characteristics and outcomes based on zone of deployment

	All-comers (n=55)	Zone 0 (n=16)	Zone 1 (n=5)	Zone 2 (n=34)
**Patient characteristics**				
Age, years	68±15	70±9	71±6	64±15
Male gender	37	12	4	21
BMI	28±6	28±5	31±7	28±5
Comorbidities				
Hypertension	49	15	4	30
Diabetes	6	1	2	3
Hypercholesterolemia	35	14	1	20
Coronary artery disease	8	4	1	3
Peripheral artery disease	2	1	0	1
Previous stroke	14	3	4	7
Chronic obstructive pulmonary disease	15	7	3	5
Hereditary thoracic aortic disease	7	1	1	5
History of open aortic surgery	31	13	4	14
**Operative outcomes**				
Technical success	55	16	5	34
Operative mortality	2	1	0	1
Stroke	0	0	0	0
Prolonged intubation >72 hrs	3	2	0	1
Renal failure requiring dialysis	1	1	0	0
Myocardial infarction	0	0	0	0
Paraparesis (temporary)	1	0	0	1
Paralysis	0	0	0	0
Retrograde type A	1	0	0	1
Aortic rupture	1	1	0	0
Access site complication	3	0	1	2
1 year mortality	5	3	1	1
Overall mortality	8	4	3	1

BMI. Body mass index. Values are mean±standard deviation or counts.
