# Supplementary material for: Initial Post-Commercialization Experience Using a Thoracic Branch Endoprosthesis: Broad Application to Real-World Patients
Source: Eur J Cardiothorac Surg. 2025 Dec 17;68(1):ezaf452. doi: 10.1093/ejcts/ezaf452 (PMC12798527; doi:10.1093/ejcts/ezaf452)

Supplementary Figure.

A. GORE TBE device; B. Zone 2 deployment with branch to LSA for adult coarctation with anomalous left vertebral artery without the need for extra-anatomic de-branching; C. Zone 1 deployment with branch to LSA in a patient who underwent LSA to LCCA bypass with ligation of the LCCA origin; D. Zone 0 deployment with branch into innominate artery in a patient who underwent manubriotomy with LCCA to RCCA transposition

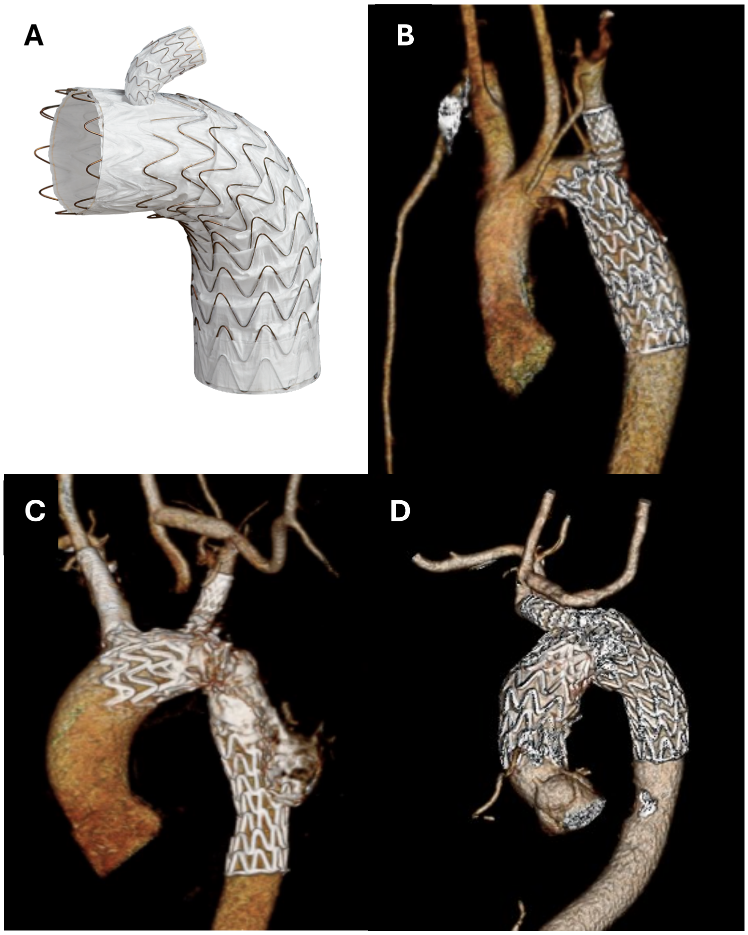

Supplement: ezaf452_Supplementary_Data [file ezaf452_supplementary_data.zip › Supplementary Figure.pdf]
